# New biomarkers for primary mitral regurgitation

**DOI:** 10.1186/s12014-015-9097-2

**Published:** 2015-09-24

**Authors:** Céline Deroyer, Julien Magne, Marie Moonen, Caroline Le Goff, Laura Dupont, Alexia Hulin, Marc Radermecker, Alain Colige, Etienne Cavalier, Philippe Kolh, Luc Pierard, Patrizio Lancellotti, Marie-Paule Merville, Marianne Fillet

**Affiliations:** GIGA Proteomic Unit, Clinical Chemistry Laboratory, University of Liège, CHU Sart Tilman, 4000 Liège, Belgium; GIGA Cardiovascular Sciences, Department of Cardiology, University of Liège, CHU Sart Tilman, 4000 Liège, Belgium; Department of Clinical Chemistry, University of Liège, CHU Sart Tilman, 4000 Liège, Belgium; GIGA-Cancer, Laboratory of Connective Tissues Biology, University of Liège, CHU Sart Tilman, 4000 Liège, Belgium; Department of Cardiovascular and Thoracic Surgery and Human Anatomy, University of Liège, CHU Sart Tilman, 4000 Liège, Belgium; Department of Biomedical and Preclinical Sciences, University of Liège, CHU Sart Tilman, 4000 Liège, Belgium; Laboratory for the Analysis of Medicines, CIRM, University of Liège, CHU Sart Tilman, 4000 Liège, Belgium

**Keywords:** Mitral regurgitation, Biomarkers, Lipid metabolism, Autophagy

## Abstract

**Background:**

Mitral regurgitation is a frequent valvular heart disease affecting around 2.5 % of the population with prevalence directly related to aging. Degeneration of mitral valve is broadly considered as a passive ongoing pathophysiological process and little is known about its physiological deregulation. The purpose of this study was to highlight new biomarkers of mitral regurgitation in order to decipher the underlying pathological mechanism as well as to allow the diagnosis and the monitoring of the disease.

**Results:**

Modulation of various blood proteins expression was examined in patients suffering from different grades of mitral regurgitation (mild, moderate and severe) compared to healthy controls. To this end, several routine clinical assays and the multi analyte profile technology targeting 184 proteins were used. High-density lipoprotein, apolipoprotein-A1, haptoglobin and haptoglobin-α2 chain levels significantly decreased proportionally to the degree of mitral regurgitation when compared to controls. High-density lipoprotein and apolipoprotein-A1 levels were associated with effective regurgitant orifice area and regurgitant volume. Apolipoprotein-A1 was an independent predictor of severe mitral regurgitation. Moreover, with ordinal logistic regression, apolipoprotein-A1 remained the only independent factor associated with mitral regurgitation. In addition, myxomatous mitral valves were studied by immunocytochemistry. We observed an increase of LC3, the marker of autophagy, in myxomatous mitral valves compared with healthy mitral valves.

**Conclusion:**

These potential biomarkers of mitral regurgitation highlighted different cellular processes that could be modified in myxomatous degenerescence: reverse cholesterol transport, antioxidant properties and autophagy.

**Electronic supplementary material:**

The online version of this article (doi:10.1186/s12014-015-9097-2) contains supplementary material, which is available to authorized users.

## Background

Mitral regurgitation (MR) is one of the most frequent valvular heart disease affecting around 2.5 % of the population with a prevalence directly related to aging [1]. Primary MR i.e. degenerative disease, rheumatic disease or endocarditis, is characterized by an impairment of the valvular apparatus whereas secondary (functional) MR is mainly due to left ventricular (LV) remodelling. Myxomatous degeneration of valve leaflets is characterised by an excessive matrix remodelling [2]. It follows leaflet enlargement and annular dilatation that impair their normal functions and lead to valve prolapse and MR [3]. So far, myxomatous degeneration was mainly considered as a passive mechanism and only a few studies investigated the underlying physiological deregulation. However, some works have highlighted interesting tracks in the understanding of the physiopathological mechanisms associated with this disease process. For example, matrix remodeling, with structural alteration of elastic fibers and collagen, could be explained by the excessive matrix metalloproteinase proteins (MMPs) and cathepsins secretion by valvular interstitial cells (VICs) in myxomatous mitral valves (MMV) [4]. These VICs can differentiate into active myofibroblasts with a significant increase of α-smooth muscle actin (α-SMA) upon TGF-β1 stimulation promoting alterations to the valve matrix architecture [5]. Other proteins like osteocalcin and low-density lipoprotein receptor-related protein 5 (Lrp5 receptor), a member of LDL family, are also up-regulated indicating an endochondral ossification process in MMV [6]. Moreover, a genetic study showed a specific mutation in the filamin-A gene causing MMV in an X-linked form of familial cardiac valvular dystrophy [7, 8]. Recently, autophagy has been emphasized as a negative mechanism that would contribute to decompensated heart failure. Indeed, in hemodynamic stress situation and pressure overload, autophagy has been showed to play a negative role in the maladaptive cardiomyocytes remodelling [9, 10]. It has been demonstrated that autophagy is induce in atrial cardiomyocytes with severe mitral and tricuspid regurgitation and that it is closely associated with the development of myolysis in this disease [11]. Therefore, it seems that the implication of autophagy becomes increasingly evident in the physiopathology of heart failure.

In order to better understand the underlying pathological mechanism of MR as well as to highlight blood biomarkers to diagnose and monitor the disease, we examined expression of various proteins in the serum of patients exhibiting MR compared to healthy controls (HC) as well as between the different grades of the disease i.e. mild, moderate and severe. To achieve this goal, we used several routine clinical assays and the multi analyte profile (MAP), a multiplex immunoassay targeting 184 proteins. Since a growing number of evidences indicate that autophagy seems to be an increasingly important actor of cardiomyocyte remodelling, we also studied its implication in MMV compared to healthy mitral valves (HMV).

## Results

### Demographic and echocardiographic data of MR patients and HC

**Fig. 1:**
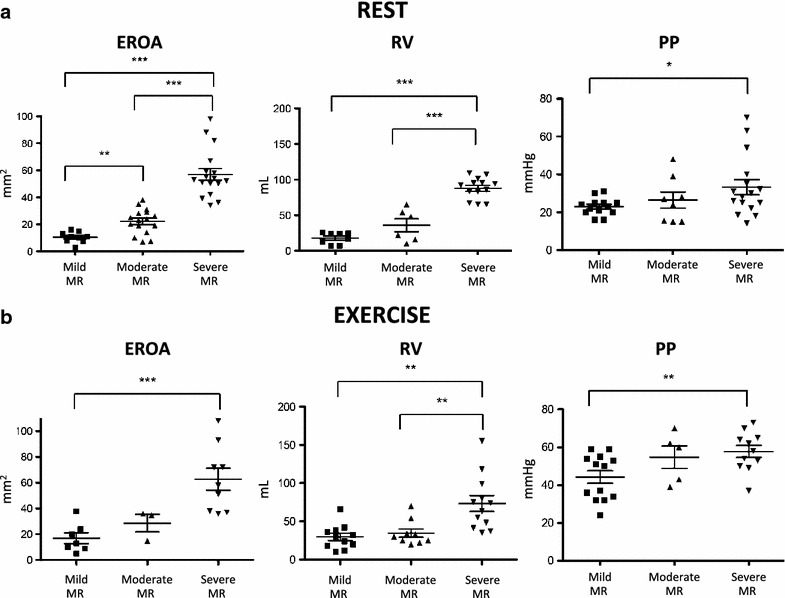
Echocardiographic data. **a** EROA, RV and PP were measured at rest on mild, moderate and severe MR patients. **b** EROA, RV and PP were then measured during exercise. These graphic representations showed that EROA remained unchanged during exercise for all MR group, that RV is slightly increased in severe MR during exercise and that PP was largely increased during exercise compared to the values at rest

The population consisted of 16 healthy controls (HC) and 64 patients affected by primary MR (cohort 1). Patients with MR were divided into three grades of MR severity determined by Doppler echocardiography: mild (EROA: 10.45 ± 4.1 mm^2^ and RV: 17.8 ± 8 mL), moderate (EROA: 22.2 ± 9.3 mm^2^ and RV: 35.8 ± 22.7 mL) and severe (EROA: 56.94 ± 17.8 mm^2^ and RV: 87.8 ± 14.9 mL). Moreover, pulmonary pressure (PP) was also measured. These data are represented in Fig. 1 at rest (A) and during exercise (B). While EROA remained mostly unchanged after exercise, we can notice a slight increase of RV for the severe group. In addition, PP was largely increased for the three MR groups upon exercise. This is in accordance with several studies including the one of Magne et al. showing that pulmonary hypertension during exercise is associated with markedly low 2-year symptom-free survival [12]. Other characteristics of the cohort are summarized in Table 1. A significant difference was found regarding the percentage of patients with cholesterol-lowering medication (HC: 12.5 %, mild: 35 %, moderate: 19 % and severe: 5 %; p = 0.018). Therefore, only for lipid assays, patients with cholesterol-lowering medication were excluded from the analysis (referred as cohort 2 in Table 2).

**Table 1:** Demographic, echocardiographic characteristics and risk factors of MR patients and healthy controls

Variables	HC (n = 16)	MR severity			p values	
		Mild (n = 23)	Moderate (n = 21)	Severe (n = 20)		
					A	B
Demographic data						
Age, years	59.44 ± 7.54	62.04 ± 10.84	61.23 ± 11.99	60.81 ± 12.85	0.91	0.92
Male sex, n (%)	8 (50)	10 (43)	12 (57)	16 (80)	0.09	0.05
Height, m	1.70 ± 0.11	1.72 ± 0.11	1.68 ± 0.08	1.71 ± 0.09	0.80	0.58
Weight, kg	77.64 ± 19.31	74.45 ± 14.85	69.56 ± 11.69	78.3 ± 13.6	0.35	0.19
Body mass index, kg/m^2^	26.36 ± 3.98	24.65 ± 3.91	24.25 ± 2.88	26.33 ± 4.21	0.26	0.22
Waist circumference, cm	95.73 ± 16.67	96.22 ± 9.91	95.5 ± 11.96	97.56 ± 12.25	0.96	0.86
Echocardiographic data						
EROA, mm^2^	–	10.46 ± 4.07	22.21 ± 9.26	56.94 ± 17.82	–	<0.0001
RV, ml	–	17.77 ± 7.99	35.83 ± 22.72	87.85 ± 14.93	–	<0.0001
PP, mmHg	–	22.92 ± 4.53	26.43 ± 12.03	33.22 ± 16.12	–	0.11
Risk factors						
Hypertension, n (%)	1 (6.25)	5 (22)	5 (24)	7 (35)	0.25	0.37
Diabetes mellitus, n (%)	1 (6.25)	2 (3)	1 (1,5)	0 (0)	0.58	0.39
Smoker, n (%)	5 (31)	1 (4)	6 (30)	3 (15)	0.05	0.03

Data are expressed as the mean ± SD except for sex, echocardiographic data, risk factors where data are expressed in number (per cent)

A: p values between the 4 groups; B: p values between the 3 MR groups

*MR* mitral regurgitation, *HC* healthy controls, *EROA* effective regurgitant orifice area, *RV* regurgitant volume, *PP* pulmonary pressure

**Table 2:** Routine clinical assays

Variables	HC	MR severity			p values	
		Mild	Moderate	Severe		
Cohort 1^a^	(n = 16)	(n = 23)	(n = 21)	(n = 20)		
Cohort 2^b^	(n = 14)	(n = 10)	(n = 16)	(n = 19)	A	B
Inflammatory markers						
CRP^a^ (mg/mL)	2.89 ± 2.69	1.50 ± 2.12	2.35 ± 3.13	1.51 ± 1.20	0.18	0.46
MPO^a^ (ηg/mL)	32.08 ± 17.71	20.83 ± 6.27	35.06 ± 30.72	41.90 ± 36.12	0.10	0.16
Heart failure markers						
BNP^a^ (pg/mL)	–	47.76 ± 35.82	73.35 ± 105.4	54.04 ± 30.83	–	0.72
Lipids						
Total cholesterol^b^ (g/L)	2.12 ± 0.38	2.25 ± 0.20	2.04 ± 0.47	2.07 ± 0.35	0.57	0.37
HDL^b^ (g/L)	0.64 ± 0.17	0.71 ± 0.06	0.61 ± 0.12	0.52 ± 0.15	0.01	0.008
LDL^b^ (g/L)	1.48 ± 1.25	1.31 ± 0.17	1.19 ± 0.43	1.24 ± 0.35	0.40	0.37
Triglycerides^b^ (g/L)	1.67 ± 1.21	1.12 ± 0.35	1.20 ± 0.48	1.56 ± 0.63	0.31	0.11
ApoA1^b^ (g/L)	1.74 ± 0.25	1.77 ± 0.17	1.63 ± 0.21	1.45 ± 0.22	0.002	0.002
ApoB^b^ (g/L)	0.99 ± 0.28	1.00 ± 0.16	0.94 ± 0.31	1.02 ± 0.27	0.75	0.60
OLDL^b^ (ηg/mL)	281.6 ± 210.2	497.2 ± 663.1	631.3 ± 830	763.2 ± 643.1	0.47	0.52
ALDL^b^ (UI/L)	390.6 ± 139.9	620.3 ± 401	707.3 ± 371	608.5 ± 402.3	0.31	0.18

Data are expressed as the mean ± SD

A: Comparisons between the 4 groups; B: Comparisons between the 3 MR groups

*MR* mitral regurgitation, *HC* healthy controls, *CRP* C-reactive protein, *MPO* myeloperoxidase, *BNP* B-type natriuretic peptide, *HDL* high-density lipoprotein, *LDL* light-density lipoprotein, *Apo* apo-lipoprotein, *OLDL* oxidized-LDL, *ALDL* antibody against OLDL

^a^Cohort 1: whole cohort

^b^Cohort 2: cohort without cholesterol-lowering medication

### Routine clinical assays

**Fig. 2:**
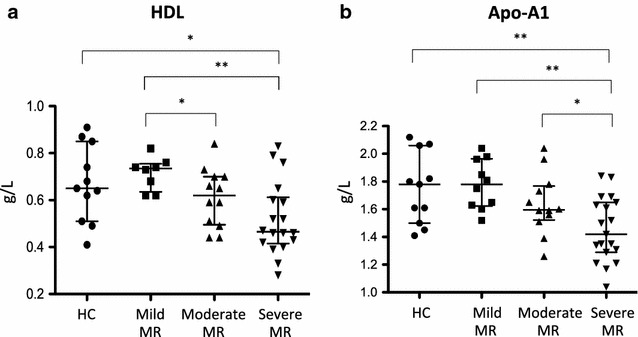
HDL and Apo-A1 levels analysis. **a** Graphic representation of HDL levels in HC, mild MR, moderate MR and severe MR. HDL levels were decreased in severe MR compared to HC (p = 0.02) and compared to mild MR (p = 0.007). HDL levels were also reduced in moderate MR compared to mild MR (p = 0.04). **b** Graphic representation of Apo-A1 levels in HC and the 3 MR groups. Apo-A1 levels were significantly reduced in severe MR compared to moderate MR (p = 0.04), mild MR (p = 0.001) and HC (p = 0.009). HDL and Apo-A1 levels were measured using enzymatic colorimetric test and nephelometry respectively on all individual samples

The different clinical assays are represented in Table 2. Inflammatory markers (CRP and MPO) levels were measured in plasma of all patients (cohort 1) but no differences between groups were detected. BNP level was not statistically different between the three MR groups. Several lipids were measured in samples from patients without cholesterol-lowering medications (cohort 2; patients with cholesterol-lowering medication were excluded from this analysis). Results are summarized in Table 2. Kruskal–Wallis test showed a significant variation of HDL level between the four groups of patients: HC (mean: 0.64 ± 0.17 g/L), mild (mean: 0.71 ± 0.06 g/L), moderate (mean: 0.61 ± 0.12 g/L) and severe (mean: 0.52 ± 0.15 g/L) with a *p* value of 0.01 and 0.008 for MR groups comparisons. Apo-A1 level was significantly lower in moderate and severe MR: HC (mean: 1.74 ± 0.25), mild MR (mean: 1.77 ± 0.17), moderate MR (mean: 1.63 ± 0.21), severe MR (mean: 1.45 ± 0.22) (p = 0.002). The lower values of HDL and Apo-A1 levels were significantly correlated with the severity of the MR (Fig. 2). Levels of these two proteins were statistically different between the HC and the severe MR group (HDL: p = 0.02 and Apo-A1: p = 0.009) and between the different grades of MR. HDL level discriminated mild MR from moderate MR (p = 0.04) or severe MR (p = 0.007). Apo-A1 level appeared significantly reduced in patients with severe MR (vs. mild MR, p = 0.001; vs. moderate MR, p = 0.04).

### Multiplex immunoassays

Human discovery MAP 175+ (Additional file 1) was performed on 20 samples composed of pools of serum from HC and MR (mild, moderate and severe) patients (cohort 1). Pools formation is described in Additional file 2. Concentration of 184 proteins was measured on each pool. 144 proteins were detected and 40 had a concentration under the detection limit. 23 proteins were statistically differentially expressed between the compared groups (*p* value <0.05) (Table 3).

**Table 3:** Proteins differentially expressed between the four comparisons (HC, mild, moderate and severe MR)

Accession number	Proteins	Genes	Significant comparisons (p value)
Up regulated proteins in MR			
P15941	Cancer Antigen 15-3	MUC1	Mild MR vs severe MR (*)
P80511	EN-RAGE	S100A12	HC vs severe MR (*)
P16860	NT-pro-BNP	NPPB	HC vs MR (**), HC vs Severe MR (**), HC vs moderate MR (**), HC vs mild MR (**)
Down regulated proteins in MR			
P02652	Apolipoprotein A-II	APOA2	HC vs MR (*), HC vs severe MR (*)
P02654	Apolipoprotein C-I	APOC1	HC vs severe MR (*)
P02749	Apolipoprotein H	APOH	HC vs MR (*), HC vs severe MR (*)
P06732/P12277	Creatin kinase MB	CKM/CKB	Mild MR vs severe MR (*)
P16581	E-selectin	SELE	HC vs MR (*), HC vs mild MR (*)
P02671/P02675/P02679	Fibrinogen	FGA, FGB, FGG	Mild MR vs severe MR (*)
P00738	Haptoglobin	HP	Mild MR vs severe MR (*), moderate MR vs severe MR (*)
P08887	Interleukin-6 receptor	IL6R	HC vs moderate MR (*)
Q14005	Interleukin 16	IL16	HC vs MR (*), HC vs moderate MR (*)
P03956	Matrix Metalloproteinase-1	MMP1	HC vs MR (*)
P09238	Matrix Metalloproteinase-10	MMP10	HC vs MR (*), HC vs moderate MR (*)
Q99616	Monocyte Chemotactic Protein 4	CCL13	HC vs MR (*), HC vs severe MR (*)
Q9HD89	Resistin	RETN	HC vs MR (*)
P02743	Serum-Amyloid-P-component	APCS	HC vs MR (**), HC vs moderate MR (*), HC vs mild MR (*)
P02787	Serotransferrin	TF	HC vs MR (*), HC vs moderate MR (*)
Q99523	Sortilin	SORT1	HC vs MR (*)
P07996	Thrombospondin-1	THBS1	HC vs MR (*), HC vs mild MR (*)
P05543	Thyroxine-Binding Globulin	SERPINA7	HC vs MR (**), HC vs severe MR (*), HC vs moderate MR (*), HC vs mild MR (*)
P02766	Transthyretin	TTR	HC vs severe MR (*), moderate MR vs severe MR (*)
P04004	Vitronectin	VTN	HC vs severe MR (*)

* p < 0.05

** p < 0.01

**Fig. 3:**
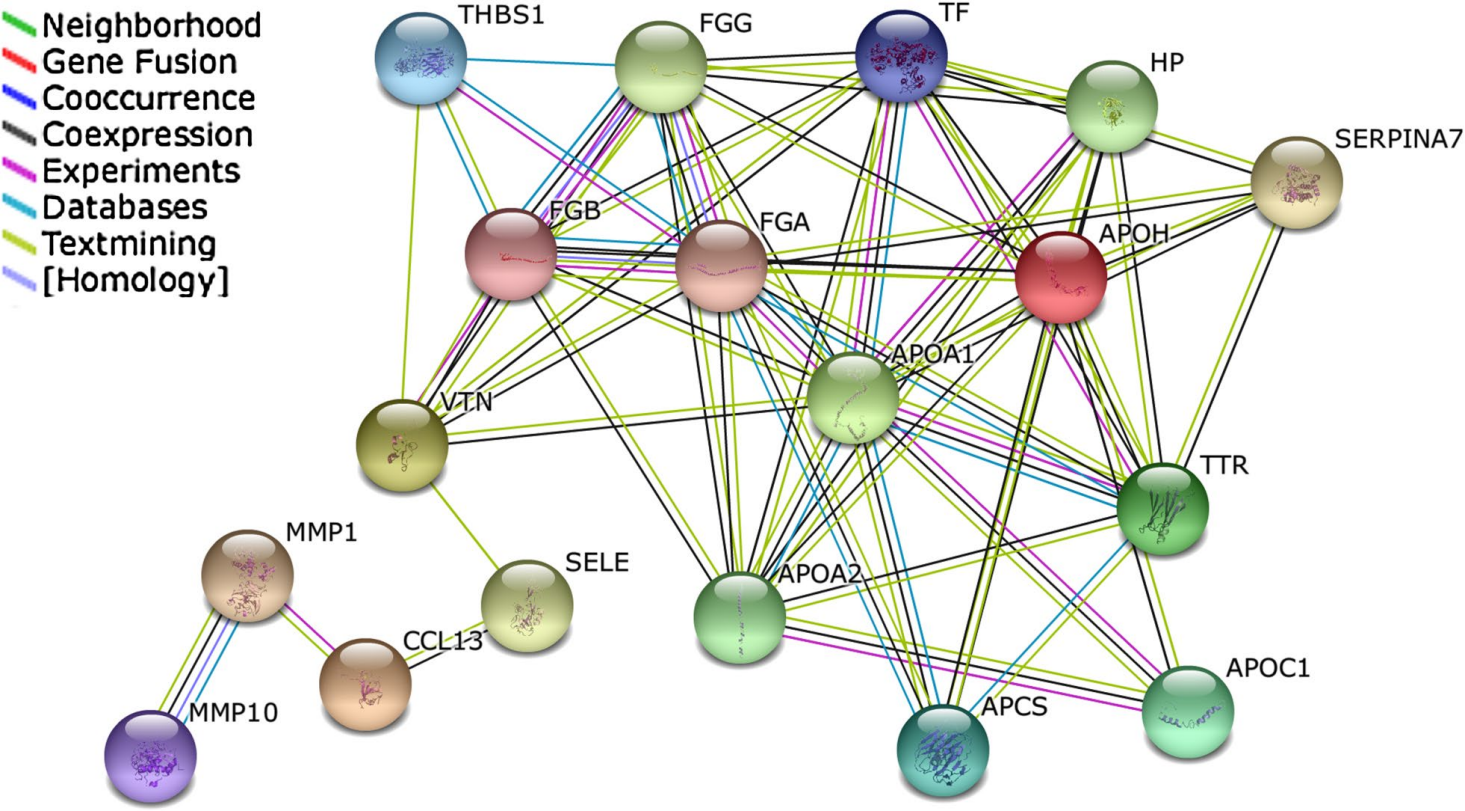
Protein interactions of potential biomarkers. (STRING software version 9.1; confidence score: 0.400 with no added interractors). Among the 24 differentially expressed proteins associated with MR, 18 were connected with each other suggesting a collaborative interaction between these potential biomarkers. *APCS* serum-amyloid-P-component, *APOA1* apolipoprotein A-I, *APOA2* apolipoprotein A-II, *APOC1* apolipoprotein C-I, *APOH* apolipoprotein H, *CCL13* monocyte chemotactic protein 4, *FGA* fibrinogen alpha-chain, *FGB* fibrinogen beta-chain, *FGG* fibrinogen gamma-chain, *HP* haptoglobin; *MMP1* matrix metalloproteinase-1, *MMP10* matrix metalloproteinase-10, *SELE* E-selectin, *SERPINA7* thyroxine-binding globulin, *TF* serotransferrin, *THBS1* thrombospondin-1, *TTR* transthyretin, *VTN* vitronectin

In order to study interactions between potential MR biomarkers, the 23 proteins of MAP and APO-A1 were uploaded in STRING software for network analysis. Note that HDL, being a lipid, could not been integrated in the analysis. Among these 24 proteins, 18 were connected with each other (Fig. 3). These data suggest collaborative interactions between these proteins highlighting different cellular processes that could be implicated in MR. Using Expasy database and Pubmed literature, we found that these proteins are implicated in main biological processes like lipid homeostasis, coagulation, autophagy and migration (Table 4).

**Table 4:** Main functions of confirmed MR biomarkers and their networked proteins

Accession number	Proteins	Genes	Functions
P02647	Apolipoprotein A-I	APOA1	Lipid homeostasis, migration, coagulation, autophagy
P02652	Apolipoprotein A-II	APOA2	Lipid homeostasis, inflammation
P02654	Apolipoprotein C-I	APOC1	Lipid homeostasis
P02749	Apolipoprotein H	APOH	Lipid homeostasis, coagulation
P16581	E-selectin	SELE	Migration, chemotaxis
P02671/P02675/P02679	Fibrinogen	FGA, FGB, FGG	Coagulation, apoptosis, vasoconstriction
P00738	Haptoglobin	HP	Coagulation, inflammation, oxidative stress
	High density lipoprotein	HDL	Lipid homeostasis, migration, autophagy, apoptosis
P03956	Matrix metalloproteinase-1	MMP1	Matrix remodeling, coagulation
P09238	Matrix metalloproteinase-10	MMP10	Matrix remodeling, migration
Q99616	Monocyte chemotactic protein 4	CCL13	Proliferation, hypoxia
P02743	Serum-amyloid-P-component	APCS	Interact with DNA and histones, associated with amyloid deposits
P02787	Serotransferrin	TF	Proliferation, coagulation
P07996	Thrombospondin-1	THBS1	Migration, proliferation, chemotaxis, apoptosis, autophagy
P05543	Thyroxine-binding globulin	SERPINA7	Coagulation, hormone transport
P02766	Transthyretin	TTRs	Coagulation, hormone transport, autophagy
P04004	Vitronectin	VTN	Coagulation, immune response, migration

**Fig. 4:**
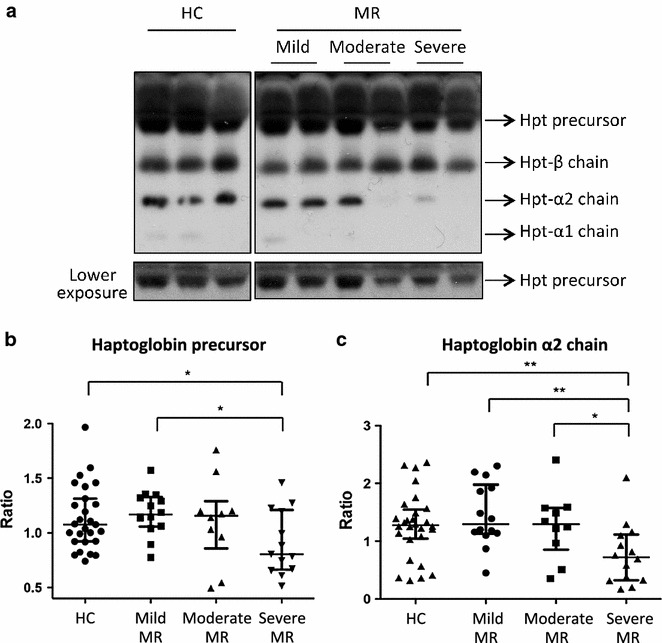
Haptoglobin level analysis. **a** Representative western blot of Hpt precursor, beta chain, alpha-2 chain and alpha 1-chain in HC and MR (mild, moderate and severe). **b** Graphic representation of Hpt precursor levels and statistical significance of comparisons between the four groups. Hpt precursor level was decreased in severe MR compared to HC (p = 0.02) and compared to mild MR (p = 0.009). **c** Graphic representation of Hpt alpha-2 chain levels and statistical significance of comparisons between groups. Hpt alpha-2 chain level was decreased in severe MR compared to HC (p = 0.004), mild MR (p = 0.002) and moderate MR (p = 0.024). Protein amounts of Hpt precursor and Hpt alpha-2 chain were quantified from western blotting using the Imagequant TL software on all individual samples

Among these different proteins, we confirmed, on all individual sera, a decrease of haptoglobin (Hpt) level by western blotting analysis. Representative image is showed in Fig. 4a. Quantification of bands intensity corresponding to the Hpt precursor by Imagequant TL software validates the differential serum level observed by MAP. Indeed level of Hpt precursor was significantly reduced in severe MR compared to HC (p = 0.02) and mild MR (p = 0.009) (Fig. 4b). Moreover, western blot analysis allowed us to quantify the intensities of the others bands corresponding to different chains of Haptoglobin (Hpt α-1, α-2 and β-chain). It appeared that level of Hpt α-2 chain was significantly lower in patients suffering from severe MR compared to the 3 other groups (p = 0.004 compared to HC, p = 0.002 compared to mild MR and p = 0.024 compared to moderate MR) (Fig. 4c).

### Factors associated with MR severity

Linear regression analysis showed that HDL and Apo-A1 levels (but not Hpt and Hpt α-2) correlated with both EROA and RV (Table 5). There was a significant negative correlation between EROA and the log transformation of HDL level (r = −0.37, p = 0.04) and of Apo-A1 level (r = −0.37, p = 0.04). Levels of HDL and Apo-A1 negatively correlated with the RV (r = −0.67, p = 0.001 and r = −0.60, p = 0.005, respectively). We also underscored that BMI and waist circumference correlated with RV (Table 5). There was a positive correlation between RV and body mass index (BMI) (r = 0.65, p = 0.003) and waist circumference (Waist circ.) (r = 0.45, p = 0.04). Finally, BMI was also positively correlated with EROA (r = 0.53, p = 0.006, Table 5). Using multivariable analysis, after adjustment for age, gender and waist circumference, we found that Apo-A1 level was independently associated with severe MR (β = 6.8 ± 2.7, p = 0.012). In addition, Apo-A1 level remained an independent predictor of severe MR even after correction for age, gender and BMI (β = 5.1 ± 2.3, p = 0.024). In a similar model using ordinal logistic regression, Apo-A1 was the only independent determinant of the severity of MR (β = 6.1 ± 2.2, p = 0.007 after age, gender and waist circumference adjustment and β = 5.4 ± 2, p = 0.004 after age, gender and BMI correction).

**Table 5:** Correlation coefficients for Apo-A1, HDL and clinical variables for MR patients

	Apo-A1	HDL	BMI	Waist circ.
EROA	−0.37*	−0.37*	0.53**	NS
RV	−0.60**	−0.67**	0.65**	0.45*

*NS* non significant

* p < 0.05; ** p < 0.01

### Autophagy activity

**Fig. 5:**
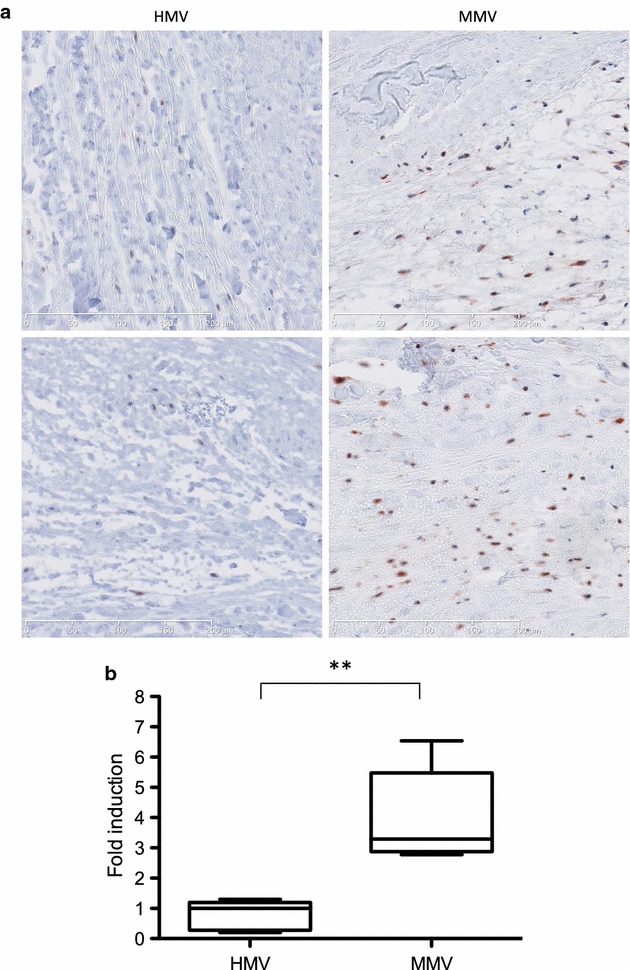
Autophagy analysis. **a** Representative IHC analysis of histological sections from HMV (*left*) and MMV (*right*) stained with anti-LC3 antibody. **b** Graphic representation of the LC3 staining relative to the valve area from HMV (n = 5) and MMV (n = 5). The incidence of LC3 puncta in mitral valve was largely increased in MMV compared to HMV (1 ± 0.48 vs 3.29 ± 1.55; p = 0.007)

Activation of autophagy process was measured in mitral valves from patients by LC3 immunohistochemistry (IHC). Autophagosome formation can be evaluated by studying LC3-II turnover, which correlates with autophagosome accumulation [14]. IHC were performed on 5 MMV and 5 HMV. Representative images are shown in Fig. 5a. The counting of LC3 punctuated structures was made on all the surface area of the valve section (including the three different layers: atrialis, spongiosa and fibrosa). As shown in Fig. 5b, the incidence of LC3-II in mitral valve was largely increase in MMV compared to HMV (1 ± 0.48 vs 3.29 ± 1.55; p = 0.007).

## Discussion

Whereas MR is a common valvular heart disease with an increased prevalence due to the population aging, little is known about its underlying physiological deregulation. This study aims to highlight underlying pathological mechanisms of MR as well as blood biomarkers reflecting the grade of the disease and allowing its diagnosis and monitoring. To this end, we compared blood protein level variations from patients suffering from MR to HC. Moreover, MR was classified as mild, moderate and severe (according to EROA and RV) (Fig. 1) allowing us to evaluate protein level variations between different grading of the disease. To do this, we used several routine clinical assays and the multi MAP technology. As the role of autophagy becomes increasingly evident in the physiopathology of heart failure, we also investigated its activity in MMV compared to HMV.

Because inflammation contributes to atherosclerosis generation, inflammatory biomarkers such as CRP and MPO are usually used to diagnose and assess the prognosis of coronary artery disease [15–18]. Therefore the potential modification of these biomarkers was investigated in patients with MR. CRP and MPO levels were not statistically different between patients with MR and HC, suggesting that inflammatory process is weak in primary MR pathology (Table 2). BNP is also widely used for the diagnosis and monitoring of various cardiovascular diseases. It allows appreciating cardiac dysfunction, which follows left ventricular remodelling [19]. BNP was measured only in MR blood samples and no difference was detected according to MR degree as previously reported (Table 2). These data are consistent with the study of Detaint et al., highlighting BNP as an emerging biomarker of the MR consequences but not of the MR degree itself [20]. However, as shown in Table 3, NT-pro-BNP was increased in all MR groups compared to HC exhibiting a decrease in cardiac function.

Lipid metabolism deregulation is well known to play a major role in coronary artery disease and in aortic stenosis [21, 22]. In our study, lipid profile was analysed in patients with MR and HC (Table 2). There was no change in the level of total cholesterol, LDL, Triglyceride, Apo-B, OLDL and ALDL. However, HDL and Apo-A1 were significantly lower in MR compared to HC. Interestingly, levels of HDL and Apo-A1 decreased according to the severity of MR (Fig. 2). Moreover, these two biomarkers gave complementary results. Indeed, HDL allowed discriminating mild from moderate or severe MR while Apo-A1 permitted to distinguish patients with severe MR from these with mild and moderate MR. HDL is well known to be inversely related to the risk of myocardial infarction [23, 24]. Its main role is to reverse cholesterol transport from peripheral tissue to the liver. Apo-A1 is the major protein component of HDL and participates in the reverse cholesterol transport by acting as a cofactor for lecithin cholesterol acyltransferase (LCAT) [25]. So, deficit in HDL and Apo-A1 levels suggest a deregulation in the reverse cholesterol transport in MR patients [26, 27].

In addition to these 2 biomarkers, MAP technology allowed us to bring out 23 potential biomarkers whose blood levels differed from MR to HC (Table 3). By western blotting and on all individual samples, we confirmed a significant decrease of Hpt and its α2-chain (Hpt-α2) levels in patients with MR (Fig. 4a); these proteins discriminated well mild to severe MR (Fig. 4b, c). Hpt-α2 also discriminated moderate MR from severe MR (Fig. 4c). Hpt is a binding partner of Apo-A1 and is associated with accumulation of atherosclerosis lesions [28, 29]. It is a plasma α2-glycoprotein synthesized by liver during acute phase. By binding to Apo-A1, Hpt prevents damages from hydroxyls radicals and allows Apo-A1 to conserve its stimulatory activity on the LCAT [30]. So, the interaction between Apo-A1 and Hpt is important for correctly handling the reverse cholesterol transport. Hpt has also been reported to have anti-oxidant and anti-inflammatory properties [31–35]. It may act as an antioxidant by preventing kidney damages. Thus, decrease of Hpt level therefore reduces its antioxidant capacity. This would be in line with different studies that highlighted an alteration of several antioxidant systems in mitral valve prolapses [13, 36]. In addition, consistent with our results, a recent proteomic study, based on iTRAQ labeling and ESI–MS/MS, showed a decrease in Hpt level of patients suffering from moderate and severe MR compared to non-MR patients [37]. However, in contrary to our study, MR severity was not considered. Indeed, as shown in Fig. 4b, c, reduction of Hpt precursor and Hpt α-2 chain levels seemed to be correlated to the disease severity. These data are also in line with another proteomic paper that recently showed a decrease of Apo-A1 and Hpt level in patient’s plasma suffering from chronic rheumatic stenosis [38].

Although protein level variations of these four proteins seem to correlate with the severity of the regurgitation, after linear regression analysis, only HDL and Apo-A1 level variations were associated with EROA and RV, which reflect MR severity (Table 5). Also note that BMI was correlated with EROA and RV whereas waist circumference with RV only. However, using multivariate analysis and after adjustment for several variables, only Apo-A1 level was independent predictor of severe MR. Moreover, with ordinal logistic regression, Apo-A1 remained the only independent determinant of MR severity.

The decrease of Apo-A1, HDL, Hpt and Hpt-α2 chain in MR patient blood mainly showed a deregulation in reverse cholesterol transport. These confirmed biomarkers were highly networked with other potential biomarkers revealed by MAP (Fig. 3). Among them, several were also implicated in lipid homeostasis (Apo-A2, Apo-C1, Apo-H). Moreover, nine proteins were implicated in coagulation (Apo-A1, Apo-H, fibrinogen, Hpt, MMP-1, serotransferrin, thyroxine-binding globulin, thansthyretin and vitronectin), six in migration (Apo-A1, E-selectin, HDL, MMP-10, thrombospondin-1 and vitronectin) and four in autophagy (Apo-A1, HDL, thrombospondin-1 and transthyretin) (Table 4).

Recently, it has been demonstrated that the induction of autophagy occurs in atrial cardiomyocytes with severe MR and that it is closely associated with the development of myolysis in this disease [11]. Therefore, autophagy activity was studied in MMV compared to HMV. The counting of LC3 punctuated structures revealed an increase of LC3-II in MMV compared to HMV (Fig. 5). This demonstrated a substantial activation of autophagy in MR patient valves. Although autophagy activation in heart diseases has been largely documented, the mechanism by which it is activated and its cellular consequences remained uncertain [9, 10]. During mild pressure overload or mild ischemia, autophagy activation can be a protective mechanism [39, 40]. Indeed, it antagonizes cardiac hypertrophy through protein degradation and eliminates misfolded proteins in order to maintain homeostasis [41]. Therefore, upregulation of autophagy in failing heart seems to be a protective response against mild hemodynamic stress. However, in more severe hemodynamic stress situation, autophagy has been showed to facilitate the maladaptive matrix remodelling [9, 10]. This has been demonstrated using mice model with a reduced activity of autophagy and where stress overload induced a lesser level of left ventricle mitral regurgitation and remodelling [42]. Inversely, in mice with high level of autophagy, pressure overload increases remodelling level [42]. Moreover, autophagy is activated in response to ROS accumulation [43]. Myxomatous degenerescence is mainly due to excessive matrix remodelling inducing abnormal accumulation of elastin, collagen and proteoglycans with an increase of ROS level [2, 5, 36, 44–46]. This could therefore suggest that autophagy is activated at the beginning of the physiopathology of MMV in response to ROS accumulation and matrix remodelling in order to eliminate excessive and toxic molecules. However, the excessive matrix remodelling found in more severe MMV let us think that autophagy is not able to act properly and on the contrary could facilitate the maladaptive matrix remodelling.

Several signalling pathways have already been identified to regulate autophagy. Among them, lipid homeostasis seems to play a significant role. Indeed, in human and mouse fibroblasts, cholesterol depletion induced autophagy activation [47]. The role of lipid metabolism in autophagy has also been demonstrated in endothelium where HDL inhibited autophagy induced by oxLDL [48]. Moreover, in endothelial progenitor cells, HDL activates cell proliferation through the PI3K/Akt pathway, which is well known to inhibit autophagy [49, 50]. Here, we highlighted a deregulation of lipid metabolism and an increase autophagy activity in MR patients.

## Conclusion

In this work, we underscored a deregulation in cholesterol transport of MR patients with a decrease of HDL, Apo-A1 and Hpt blood levels. We also showed an increase of autophagy activity in MMV that could be related to HDL and Apo-A1 low levels. All these results show a potential disruption of lipid metabolism and autophagic activity in the MR physiopathology. This provides new information in the understanding of myxomatous degeneration and offers new insight in the development of therapeutic strategies to improve valve leak. In addition, these differentially abundant proteins could be considered as biomarkers for the diagnosis and the monitoring of the disease. Nevertheless, a validation study on a larger cohort is required. The well-established cardio-protective nature of HDL and Apo-A1 made them targets for cardiovascular therapies aimed to raise circulating levels. Currently, no specific drug is used to improve valve leak. The only effective treatment is to repair or replace the valve. Therefore, the use of a specific medication against MR is attractive. For example, fibrates, niacin or statins can increase HDL circulating levels. Moreover, novel class of drug like cholesterol-ester-transport-protein inhibitors (CETP) or intravenous Apo-A1-therapy, up-regulators of endogenous Apo-A1 production and Apo-A1 mimetic peptides deserve to be evaluated for MR treatment.

## Methods

### Blood sample collection

Blood samples were collected from MR patients and healthy controls (HC). They were enrolled from 2009 to 2012 at the “Centre Hospitalier Universitaire de Liège”. Patients were prospectively seen in our Heart Valve Clinic and included in the study when inclusion/exclusion criteria were fulfilled. MR groups only comprise patients suffering from primary MR. Patients with other valvular diseases (i.e. secondary MR, aortic, tricuspid or pulmonary stenosis or regurgitation) were excluded from the MR group. HC included peoples without valvular diseases. A total of 80 serum and plasma samples from patients with primary MR and from HC were collected into vacutainer tubes at the time of echocardiography measurement. Between 30 min and 2 h after collection, blood was centrifuged at 3000*g* for 10 min to collect serum and plasma and immediately processed for analysis or aliquoted and frozen at −80 °C until use.

Patients affected by MR were classified into three categories according to the severity of the pathology [defined by the effective regurgitant orifice area (EROA) and regurgitant volume (RV) values]: mild (EROA <20 mm^2^, RV <30 mL), moderate (EROA: 20–39 mm^2^, RV 30–59 mL), and severe (EROA >40 mm^2^, RV >60 mL). The ethical committee of the University hospital (CHU-Liège) approved this study and all patients gave their written informed consent.

### Echocardiographic method

Systolic pulmonary arterial pressure was derived from the peak systolic velocity of the tricuspid regurgitant jet according to the simplified Bernoulli equation and adding 10 mmHg for the right atrial pressure, as previously validated [12]. Echocardiographic investigations were performed with a Vivid 7 or 9 imaging device (GE Healthcare, Little Chalfont, UK). All echocardiographic parameters were averaged over three cardiac cycles.

The severity of MR was assessed as recommended by current guidelines (Lancellotti et al., EHJ CV imaging, 2013) and using an integrative approach. EROA, a marker of the valvular lesion and RV, a marker of volume overload, were quantified using the proximal isovelocity surface area (PISA) method. Only one physician read all echocardiograms.

### Routine clinical assays

CRP (C-reactive protein) level was measured using an immunoturbidimetric test (Roche Diagnostics, Manheim, Germany) (reference values: 1–6 mg/L). MPO (myeloperoxidase), OLDL (oxidized-LDL) and ALDL (Anti-OLDL) levels were measured with ELISA kit from Biomedica (Wien, Austria) and Mercodia (Ippsala, Sweden) respectively (reference values: <55 ng/mL; 200–600 UI/L; <500 ng/mL respectively). Brain natriuretic peptide (BNP) level was measured by an immunofluorescence assay (Biosite, Beckman Coulter, San Diego, LA, USA). Total cholesterol (reference values: <1.9 g/L); HDL (high density lipoprotein) (reference values: >0.4 g/L for male and >0.5 g/L for female); LDL (light density lipoprotein) (reference values: <1.15 g/L) and triglycerides (reference values: <1.5 g/L) were measured using enzymatic colorimetric test from Roche diagnostics. Apo (Apolipoprotein) A1 and B were measured by nephelometry using antibodies (Siemens, Erlangen, Germany) (reference values: 1.05–2.05 g/L and 0.6–1.3 g/L respectively).

### Multiplex immunoassay: multi-analyte profile (MAP)

Multi-analyte profile (MAP) technology is a commercially available high-throughput assay from Myriad RBM (Austin, Texas, United States). The Human discovery MAP175+ consisted of 184 predefined proteins allowing covering dozens of different pathways. These 184 proteins are listed on Additional file 1. Concentration determination of these predefined proteins was performed on 20 pooled serum samples from patients suffering from mild, moderate and severe MR and from healthy controls (HC) (Additional file 2). MAP technology is based on the classic capture-sandwich method using capture antibodies attached to fluorescently encoded microspheres. Proteins concentration was determined by the flow cytometer Luminex machine.

### Protein interactions analysis

Protein interactions network was built using the software STRING version 9.1 (string-db.org/). STRING is an online database enabling to predict physical and functional protein–protein interactions based on databases and literature. In Fig. 3, the confidence score was set at 0.400 with no added interactors. Biological functions study of various proteins was made using Expasy database and Pubmed literature.

### Western blot analysis of haptoglobin

Equal volumes of each serum sample from HC and MR patients (mild, moderate and severe) (n = 80) were loaded on sodium dodecyl sulfate (SDS)-polyacrylamide gel. Separated proteins were then transferred on polyvinylidene difluoride membranes (PVDF). Membranes were blocked in a 5 % milk solution and then incubated with the polyclonal anti-Haptoglobin antibody (Abcam, Cambridge, UK). After washing, membranes were incubated with the anti-chicken secondary antibody (Abcam). The revelation was performed using the enhanced chemiluminescence detection reagent (ECL kit, Thermo Scientific, MA, USA). The detected signals were analysed by densitometry and the intensity of each band was measured with the Imagequant TL software (GE Healthcare). To normalize protein levels, the value of the band corresponding to each protein level was divided by the band intensity of the intergel controls (i.e. the same sample loaded twice on each gel).

### Tissue collection

P2 segments of posterior leaflets from MMV (n = 5) were obtained during valvuloplasty to correct severe mitral regurgitation. Normal P2 segments of posterior leaflets from HMV (n = 5) were obtained from hearts of donors rejected for transplantation. Tissues collection was made in collaboration with the Laboratory of Connective Tissue Biology (GIGA-Cancer, ULg) and the Department of Cardiovascular and Thoracic Surgery and Human Anatomy (CHU Sart-Tilman, ULg) as previously described [13]. The protocol was conformed to the principles outlined in the Declaration of Helsinki and was approved by the Ethics Committee of Liège University Hospital. All patients gave their written informed consent.

### Immunohistochemistry

Briefly, a 2 mm strip of tissue was cut in the median of the P2 leaflet from the free edge to the annulus. The strips were fixed in 4 % neutralized paraformaldehyde (Sigma-Aldrich) for 2 h, dipped in 70 % (v/v) ethanol and embedded in paraffin. Immunohistochemistry was performed on valvular tissue sections after dewaxing and citrate unmasking using a primary monoclonal antibody against LC3 (1/200, Cell signaling). LC3 was revealed with a secondary biotinylated goat anti-rabbit antibody (1/400, Dako) and an HRP/streptavidin solution (Dako). Staining was revealed with software OlyVIA (Olympus) and Quantity One 4.6 (BioRad). The number of LC3 punctuated structures was counted blindly with the ImageJ software (NIH) on the complete tissue section of the strips.

### Statistical analysis

All data are presented as median ± SD or percentage. Comparisons between two groups were assessed using the nonparametric Mann–Whitney Test. Normality of our data was assessed by the Kolmogorov–Smirnov normality test. All data were normally distributed (p > 0.05) excepting HDL, Apo-A1 and RV (p < 0.05). For comparisons between more than two groups, one-way ANOVA or Kruskal–Wallis test were used as appropriate. Correlations between data were evaluated with linear regressions (Pearson or Spearman test as appropriate). For correlation between abnormally and normally distributed values, abnormally distributed values were transformed in logarithm. Logistic regression and ordinal logistic regression analysis were used to identify independent predictors of severe MR (i.e. vs. both mild and moderate MR) and MR grade severity (i.e. mild vs. moderate vs. severe), respectively. Age, gender and body mass index (BMI) or waist circumference were entered as adjustment variables in all multivariable models.

For immunohistochemistry, a mean value of LC3 punctuated structures blindly counted using ImageJ software (NIH) was established relatively to the total surface of the section and expressed as a fold induction relative to the control condition (i.e. HMV) taken as 1.

p values <0.05 were considered statistically significant.

## Additional files

**Additional file 1:** Human discovery MAP175+ (Myriad RBM).
**Additional file 2:** Samples pool for MAP.

## Abbreviations

MR: mitral regurgitation
LV: left ventricular
MMPs: matrix metalloproteinase proteins
VICs: valvular interstitial cells
MMV: myxomatous mitral valves
α-SMA: α-smooth muscle actin
HC: healthy controls
MAP: multi analyte profile
HMV: healthy mitral valves
HC: healthy controls
EROA: effective regurgitant orifice area
RV: regurgitant volume
CRP: C-reactive protein
MPO: myeloperoxidase
OLDL: oxidized-LDL
ALDL: anti-OLDL
BNP: brain natriuretic peptide
HDL: high density lipoprotein
LDL: light density lipoprotein
Apo: apolipoprotein
PP: pulmonary pressure
Hpt: haptoglobin
BMI: body mass index
IHC: immunohistochemistry
LCAT: lecithin cholesterol acyltransferase
