# Supplementary material for: New biomarkers for primary mitral regurgitation
Source: Clin Proteomics. 2015 Sep 24;12:25. doi: 10.1186/s12014-015-9097-2 (PMC4581160; doi:10.1186/s12014-015-9097-2)
Supplement: Supplementary file 1 — Additional file 1: Human discovery MAP175+ (Myriad RBM). [file 12014_2015_9097_MOESM1_ESM.docx]

**S1 (1):** Human discovery MAP175+ (Myriad RBM)

| 6Ckine | Cancer Antigen 19-9 | Fibroblast Growth Factor 4 |
| --- | --- | --- |
| Adiponectin | Carcinoembryonic Antigen | Fibroblast Growth Factor basic |
| Agouti-Related Protein | CD40 Antigen | Follicle-Stimulating Hormone |
| Alpha-1-Antichymotrypsin | CD40 Ligand | Glucagon |
| Alpha-1-Antitrypsin | CD5 Antigen-like | Glucagon-like Peptide 1, active |
| Alpha-1-Microglobulin | Chemokine CC-4 | Glucagon-like Peptide 1, total |
| Alpha-2-Macroglobulin | Chromogranin-A | Glutathione S-Transferase alpha |
| Alpha-Fetoprotein | Ciliary Neurotrophic Factor | Granulocyte Colony-Stimulating Factor |
| Amphiregulin | Clusterin | GranulocyteMacrophageColonyStimuFactoR |
| Angiopoietin-2 | Complement C3 | Growth Hormone |
| Angiotensin-Converting Enzyme | Complement Factor H – Related Protein 1 | Growth-Regulated alpha protein |
| Angiotensinogen | Cortisol | Haptoglobin |
| Apolipoprotein A-I | C-Peptide | Heat Shock Protein 60 |
| Apolipoprotein A-II | C-Reactive Protein | Heparin-Binding EGF-Like Growth Factor |
| Apolipoprotein A-IV | Creatine Kinase-MB | Hepatocyte Growth Factor |
| Apolipoprotein B | Cystatin-C | Human Chorionic Gonadotropin beta |
| Apolipoprotein C-I | EN-RAGE | Immunoglobulin A |
| Apolipoprotein C-III | Eotaxin-1 | Immunoglobulin E |
| Apolipoprotein D | Eotaxin-3 | Immunoglobulin M |
| Apolipoprotein E | Epidermal Growth Factor | Insulin |
| Apolipoprotein H | Epidermal Growth Factor Receptor | Insulin-like Growth Factor Binding Protein 2 |
| Apolipoprotein(a) | Epiregulin | Intercellular Adhesion Molecule 1 |
| AXL Receptor Tyrosine Kinase | Epithelial-Derived Neutro-Activat Prot 78 | Interferon gamma |
| B cell-activating factor | E-Selectin | Interferon gamma Induced Protein 10 |
| B Lymphocyte Chemoattractant | Factor VII | Interferon-inducible T-cell α chemoattractant |
| Beta-2-Microglobulin | Fas Ligand | Interleukin-1 alpha |
| Betacellulin | FASLG Receptor | Interleukin-1 beta |
| Brain-Derived Neurotrophic Factor | Fatty Acid-Binding Protein, heart | Interleukin-1 receptor antagonist |
| Calbindin | Ferritin | Interleukin-2 |
| Cancer Antigen 125 | Fetuin-A | Interleukin-3 |
|  | | |
| **S1 (2):** Human discovery MAP175+ (Myriad RBM) | |  |
| Cancer Antigen 15-3  Interleukin-5 | Fibrinogen  Matrix Metalloproteinase-10 | Interleukin-4  Sex Hormone-Binding Globulin |
| Interleukin-6 | Monocyte Chemotactic Protein 1 | Sortilin |
| Interleukin-6 receptor | Monocyte Chemotactic Protein 2 | Stem Cell Factor |
| Interleukin-7 | Monocyte Chemotactic Protein 3 | Stromal cell-derived factor-1 |
| Interleukin-8 | Monocyte Chemotactic Protein 4 | Superoxide Dismutase 1, soluble |
| Interleukin-10 | Monokine Induced by Gamma Interferon | T Lymphocyte-Secreted Protein I-309 |
| Interleukin-12 Subunit p40 | Myeloid Progenitor Inhibitory Factor 1 | Tamm-Horsfall Urinary Glycoprotein |
| Interleukin-12 Subunit p70 | Myeloperoxidase | T-Cell-Specific Protein RANTES |
| Interleukin-13 | Myoglobin | Tenascin-C |
| Interleukin-15 | Nerve Growth Factor beta | Testosterone, Total |
| Interleukin-16 | Neuronal Cell Adhesion Molecule | Thrombomodulin |
| Interleukin-17 | Neuron-Specific Enolase | Thrombospondin-1 |
| Interleukin-18 | Neutrophil Gelatinase-Associated Lipocalin | Thyroid-Stimulating Hormone |
| Interleukin-23 | NT-proBNP | Thyroxine-Binding Globulin |
| Kidney Injury Molecule-1 | Osteopontin | Tissue Inhibitor of Metalloproteinases 1 |
| Lectin-Like Oxidized LDL Receptor 1 | Pancreatic Polypeptide | TNF-Related Apoptosis-Inducing Lig Rec 3 |
| Leptin | Peptide YY | Transforming Growth Factor alpha |
| Luteinizing Hormone | Placenta Growth Factor | Transforming Growth Factor beta-3 |
| Macrophage Colony-Stimulating Factor 1 | Plasminogen Activator Inhibitor 1 | Transthyretin |
| Macrophage inflammatory protein 3 beta | Platelet-Derived Growth Factor BB | Trefoil Factor 3 |
| Macrophage Inflammatory Protein-1 alpha | Progesterone | Tumor Necrosis Factor alpha |
| Macrophage Inflammatory Protein-1 beta | Proinsulin, Intact | Tumor Necrosis Factor beta |
| Macrophage Inflammatory Protein-3 alpha | Proinsulin, Total | Tumor Necrosis Factor Receptor 2 |
| Macrophage Migration Inhibitory Factor | Prolactin | Vascular Cell Adhesion Molecule-1 |
| Macrophage-Derived Chemokine | Prostate-Specific Antigen, Free | Vascular Endothelial Growth Factor |
| Malondialdehyde-Modif Low-Density Lipo | Pulmonary & Activat-Reg Chemokine | Vitamin D-Binding Protein |
| Matrix Metalloproteinase-1 | Rec for advanced glycosylation end products | Vitamin K-Dependent Protein S |
| Matrix Metalloproteinase-3 | Resistin | Vitronectin |
| Matrix Metalloproteinase-7 | S100 calcium-binding protein B | von Willebrand Factor |
| Matrix Metalloproteinase-9 | Serotransferrin |  |
| Matrix Metalloproteinase-9, total | Serum Amyloid P-Component |  |
