# Supplementary material for: New biomarkers for primary mitral regurgitation
Source: Clin Proteomics. 2015 Sep 24;12:25. doi: 10.1186/s12014-015-9097-2 (PMC4581160; doi:10.1186/s12014-015-9097-2)
Supplement: Supplementary file 2 — Additional file 2: Samples pool for MAP. [file 12014_2015_9097_MOESM2_ESM.docx]

**S2:** samples pool for MAP

|  | **HC** | | | | |
| --- | --- | --- | --- | --- | --- |
|  | **POOL 1** | **POOL 2** | **POOL 3** | **POOL 4** | **POOL 5** |
| N (number) | 4 | 3 | 3 | 3 | 3 |
| Age (years) | 59 | 60 | 60 | 59 | 60 |
| % Men | 50 | 33 | 66 | 33 | 66 |
| % Women | 50 | 66 | 33 | 66 | 33 |
|  | **MR Mild** | | | | |
|  | **POOL 6** | **POOL 7** | **POOL 8** | **POOL 9** | **POOL 10** |
| N (number) | 5 | 5 | 5 | 4 | 4 |
| Age (years) | 61 | 63 | 62 | 63 | 61 |
| % Men | 40 | 40 | 40 | 50 | 50 |
| % Women | 60 | 60 | 60 | 50 | 50 |
|  | **MR Moderate** | | | | |
|  | **POOL 11** | **POOL 12** | **POOL 13** | **POOL 14** | **POOL 15** |
| N (number) | 5 | 4 | 4 | 5 | 3 |
| Age (years) | 60 | 62 | 62 | 61 | 59 |
| % Men | 69 | 50 | 50 | 60 | 66 |
| % Women | 31 | 50 | 50 | 40 | 34 |
|  | **MR Severe** | | | | |
|  | **POOL 16** | **POOL 17** | **POOL 18** | **POOL 19** | **POOL 20** |
| N (number) | 5 | 4 | 4 | 4 | 3 |
| Age (years) | 60 | 61 | 61 | 62 | 61 |
| % Men | 80 | 75 | 75 | 75 | 100 |
| % Women | 20 | 25 | 25 | 25 | 0 |
